# Annotations

**Published:** 1889-03-23

**Authors:** 


### Annotations.

**Growing Fat.**

It is not well to be too fat, as it is not well to be too lean. Men generally recognize that youth is over when the weight begins to increase unduly, and the waistband becomes too tight. Horses do not lose their agility when they approach middle age, and for that reason they do not get too fat. A horse of nine or ten is at his very best for hunting or coaching purposes if he has been well used. But a horse of ten is decidedly a middle-aged animal. For all kinds of work middle-age ought to be the best period with man; but it is not generally so for active outdoor work, because most of us are too fat and puffy. Some people cannot prevent themselves getting unwieldily stout, but others can. Perhaps the most common cause of the too great accumulation of fat in human beings is "deficient oxidation." And what is deficient oxidation? The elements of which the body is composed require for their health a certain quantity of oxygen; and those parts that have reached the stage of waste require a good deal. That oxygen can ordinarily be obtained from one source only—the atmospheric air; and by one means only—transmission by the blood. Failing oxidation and the removal of waste, certain products which are in course of conversion into waste became converted into fat instead; and whereas they ought to be got rid of as cumberers of the ground, they are shut up in different parts of the body, and become permanent obstructives to physiological processes. Oxidation then is an exceedingly important function of the body. It is possible to retard, and also to accelerate it. When it is retarded fat is usually one of the consequences. In order to accelerate it, two things are indispensable—great bodily activity and an abundant supply of pure air. Every time the heart beats, a definite quantity of atmospheric air is passed into the lungs, and from them the oxygen is transmitted by the blood streams direct to the tissues. If the heart beats rather feebly seventy times a minute a certain quantity of oxygen will be carried to the tissues; but if it beat strongly eighty times a minute, it is obvious that much more will of necessity be carried. Similarly, if nearly 99 parts of atmospheric air in 100 consist of oxygen, other things being equal, the blood will be well oxidised; but if only 92 or 93 parts be oxygen, it is equally clear that the blood will be ill-oxidised. Under these latter circumstances we are all liable to become very fat, and to suffer many evils of which the fatness is but an index and outward sign. Walking, running, leaping, wrestling, tricycling, and other such exercises are the enemies and destroyers of fat. If these be carried out thoroughly in a pure atmosphere, very few middle-aged persons will need to complain of their stoutness. Laziness and indoor living are the parents of physical rotundity and intellectual dulness.

**How to "Cane" Boys.**

An important practical question has been raised at the Worship Street Police Court. Mr. Bushby considers that "caning" a schoolboy on the hand is improper; and a short time ago he convicted Mr. Gardner, head master of the Church Street Board School, Bethnal Green, for giving a boy four "handers," or "palmies," as they are called in the North. The School Board authorities by no means agree with the Worship Street magistrate, and desire to get a decision from the High Court as to what is, and what is not a proper punishment for boys. There is no doubt that the question is one of daily moment to schoolmasters. It is certain that if discipline is to be maintained, punishment must sometimes be resorted to. But if the master who punishes can only do so at the risk of conviction and fine, or even worse, it is more than likely that punishment, and with it "school discipline," will often be allowed to "go by the board." The real point at issue is this: To what part of the body may strokes with a birch or cane be applied, without risk of injury to important structures? All persons, both schoolmasters and others, are now agreed that striking the head or face, or boxing the ears, is dangerous. The practice has indeed to a large extent been given up, except in country villages, where heads are dense and nerves are not sensitive. It ought to be given up universally. "Handers," or "palmies," are of much earlier origin than either Board or National schools. It is not improbable that they were known to the Ancient Britons, or even to the builders of the Tower of Babel. As administered in country schools thirty years ago, with a piece of thin wood, three inches broad and two feet long, the stroke was delivered upon the four prominent ridges of the palm, and seldom or never produced any injurious results. But as now given, with a round, thin cane, it becomes a "cutting" stroke, and if it fall with sharp energy across the centre and hollow of the palm of soft-handed town children may do serious mischief, not only to the skin, but to important structures beneath. In all large towns, therefore, "handers" are dangerous, and should be discontinued. In country districts, where rough occupations make the integument thick and hard there is less risk. There remain for consideration a "thrashing across the shoulders," and a good sound "birching." From a medical and scientific standpoint "birching" is undoubtedly the most suitable and the safest form of punishment a boy can receive. The preparation for it requires time, and allows the anger of the teacher to subside a little. That is an undoubted advantage. The part birched is sufficiently sensitive to make the punishment decidedly "smart" in every sense. The painful effect by no means ceases with the last swish of the cane, but remains for a considerable time, making ease and idleness alike unpleasant. Finally, no harm can be done unless the bircher be of such a savage temper as ought to ensure his permanent dismissal from the service of school committees. Some people think birching indecent. Those poor creatures ought to have had human bodies specially made for themselves on a different pattern from that after which they are at present constructed. If there be any indecency, it is in their own ridiculous minds. For disciplinary purposes in boys' schools there is no other punishment which is at once so efficacious, so safe, and so properly humiliating as a "good sound birching."

**Retribution.**

Men with reasonable minds are accustomed to pay a good deal of attention to the ways of Nature, and they learn many valuable lessons in her school. Among other things they find that though she is very benevolent to those who are industrious, sensible, and docile, she has small patience with the idler, the self-willed and the foolish. She punishes them without scruple, and without hesitation, by means of such strong measures as starvation, inflammation of the lungs, brain fever, and other such apparently heartless and cruel ways. Soft and tender souls may cry out against her "cruelty," or even break their hearts in some cases; but Nature never makes any change in her laws on that account. She insists upon one of two things—reasonable obedience or stern and inexorable punishment. We call the attention of the well-meaning people of the present day, who are all for tenderness towards all sorts of criminals, to these undoubted ways of our inflexible Tutoress. For all repentant criminals, let there be a place of penitence and help on the pathway of virtue; though even repentant criminals will best show their penitence by sorrow for their crimes and willingness to endure reasonable punishment. For unrepentant criminals, punishment swift, sure, and severe is the merited doom. Last week, at Liverpool, Mr. Justice Charles passed sentence on three roughs, who had been convicted of robbing a woman, with violence. Those three men attacked one women, and, not content with robbing, brutally illtreated her. The judge said there was only one way of dealing with such fellows. Not only must they go to prison for six months, but each of them must, in addition, receive fifteen lashes with the "cat." The study of the sciences bearing upon medicine has this advantage: that it compels the mind to see and acknowledge the stern and unbending laws of retribution. Do wisely and well, say those sciences, and your reward will follow. Do foolishly and ill, and your punishment will not tarry. The gentle and sensitive souls who believe in sugar plums for hardened criminals, should study Nature's ways for a few years with candid impartiality. They would then know that, under the reign of law at any rate, punishment should always, as far as possible, be commensurate with crime. He who deprives another of his money must have his own money, or its equivalent in time, taken away by adequate imprisonment; he who resorts to blows and brutality must in turn be visited with blows as severe and painful as those he inflicted. Nature and common sense agree in the doctrine of righteous retribution.

